# Genetic counseling prior to Assisted Reproductive Technology procedures in the era of cytogenomics

**DOI:** 10.5935/1518-0557.20180050

**Published:** 2018

**Authors:** Roberto Coco

**Affiliations:** 1 Fecunditas - Instituto de Medicina Reproductiva afiliado a la UBA de Argentina

**Keywords:** genetic counseling, assisted reproductive technologies, genetic testing, NGS, PGT-A, PGT-M

## Abstract

The possibility of sequencing hundreds of genes simultaneously and performing molecular karyotyping thanks to the introduction of novel genetic tools has expanded the use of preconception screening for blastocyst recessive mutations and aneuploidies before embryo transfer, with the ultimate purpose of increasing the proportion of normal healthy newborns. Since medically-assisted reproduction procedures are increasingly required to be eugenic, and the aforementioned genetic tests cover only half of the potential genetic diseases occurring at birth, it seems reasonable to incorporate genetic counseling in the practice of assisted reproduction to avoid prosecution for malpractice.

Congenital defects are structural and functional anomalies that may cause different grades of disability and even the death of affected individuals. They are present from the early stages of formation and are considered developmental anomalies. These anomalies are caused by a number of reasons, including chromosomal, multifactorial, monogenic, and teratogenic factors. Chromosome abnormalities are more prevalent at the start of pregnancy, produce more than 50% of first-trimester miscarriages, and are found in a third of the fetuses with major malformations detected by ultrasound in the second trimester. About 3% of newborns have major malformations. In Argentina, these constitute the second cause of infant mortality and are responsible for 30% of hospitalizations in pediatric hospitals, a reality shared by other Latin American countries. Couples of reproductive age have a 3% chance of having an abnormal child, and chances may increase depending on disorders occurred during adulthood or later.

Pregnancy planning, preconception consultation, and timely detection of risk factors help prevent birth defects. The knowledge and dissemination of risk factors such as drug and alcohol abuse, as well as the prevention and treatment of maternal infections and the control of diseases such as diabetes, comprise the fundamental pillars of birth defect prevention. On the other hand, adequate and sufficient diet and folic acid supplementation have been shown to be useful in reducing the occurrence and recurrence of neural tube defects.

In the imaginary of couples with access to assisted reproductive technology - and to the possibility of checking the normal development of the fertilized oocyte - there is the belief that all births culminate in normal babies. Undoubtedly, the medical responsibility of the team assisting the patient from the beginning of pregnancy is greater than that of the obstetricians offering care to women who became pregnant without medical help.

And without a doubt, in contexts of ART the biomedical team has ample opportunity to plan pregnancies and minimize gestational and neonatal risk. In the early days of in vitro fertilization, there was fear that the first stages of in vitro development might be mutagenic. However, increases in *de novo* dominant or recessive mutations were not seen, and specialists in the area were reassured and excited with the prospects of assisted reproduction programs. Some seven million babies have been born with the aid of assisted reproductive technology. Although most are healthy, an increase of 30-40% in births with congenital malformations has been estimated (Davies *et al*., 2012; Ericson & Källén, 2001; Farhangniya *et al*., 2013; Farhi *et al*., 2013; Hansen *et al*., 2012; Källén et al., 2005; Tararbit *et al*., 2013; Lacamara *et al*., 2017; Massaro *et al*., 2015). However, there are no systematic epidemiological studies confirming increased risk of genetic diseases detected at birth, during the course of one's life or occurring in their descendants.

The importance of genetics in reproductive practice begins with the implementation of chromosome testing in the 1960s, a long time before the advent of in vitro fertilization. Important authors, including Anne Chandley (Chandley, 1983), posited that practically every genetic disorder might directly or indirectly interfere with fertility, and that before attempting any therapeutic procedure to reverse infertility, one should rule out the existence of genetic causes, because infertility might be a selection mechanism devised to prevent malformations. Nevertheless, as reproductive technology demonstrated that infertility might be reversed, genetic tests for infertile couples have been abandoned in poorer countries to diminish costs. Medicine is definitely becoming more predictive, but the lack of allocation of resources in our region makes predictive medicine a distant reality.

In international congresses of societies related to reproduction and genetics, attention has been given to preconception and preimplantation genetic tests. Preconception tests are designed to find recessive mutations, while preimplantation tests are used to select blastocysts without aneuploidies. Genetic tests performed with the DNA of peripheral blood leukocytes or oral mucosa swabs do not rule out the existence of germ cell mutations. Therefore, it must be emphasized that tests with normal results only downgrade the potential genetic risk present in all newborns. The Perruche case [Fr. Cour de Cassation, Assemblée pléniére, 2000] tried at the beginning of the century, established jurisprudence in France on disabled children's right not to be born. Since then, lawsuits involving children with congenital disorders have become more frequent, particularly when they were conceived with medical help.

At first, assisted reproduction technologies were condemned because they were considered eugenic, while today they might stand trial for not being eugenic enough. Eugenics has become a requirement in assisted reproduction, and noncompliance might be interpreted as medical malpractice. An example that comes to mind is the recent case brought against an Argentinian assisted reproduction center accredited by RedLara, in which an embryo donation was performed (Rivara lawsuit, 2015). Donor semen samples were evaluated for 29 cystic fibrosis mutations and were negative for all of them. The female donor was evaluated only for the Delta F 508 mutation, one of the most common in patients affected by cystic fibrosis. Since the donor semen did not carry any of the tested mutations, the female donor did not have to be tested for the same mutations, because her carrying some of them did not imply risk of having a baby with cystic fibrosis. A molecular test performed on the baby showed it carried mutation G542X. Given that the donor semen did not have this mutation, it was inferred that the female donor must have transmitted it. But for the child to be affected there must be a pair of mutations for the same gene. If the female donor transmitted mutation G542X, the non-identified mutation must have come from the semen used. However, the semen bank was dismissed from carrying out tests for a greater number of mutations, while the center that delivered the treatment and selected the donor was found guilty by the judges for medical malpractice, since they did not carry out the same tests performed with the donor semen; an incredible, yet true story.

As "the right to be born healthy and normal" and "the greater medical responsibility" acquire more importance in the practice of assisted reproduction with patient-own or donor gametes, it is almost mandatory, in times of genomics, to inform patients of how they can minimize the genetic risks to which they might be exposed, even if they do not face significant risk on account of family history or ethnicity.

Screening for recessive mutations, "carrier testing," and "preimplantation genetic diagnosis" to screen for blastocyst aneuploidies - PGT-A or PGS - have been widely used and regarded as well-accepted procedures by physicians and patients. PGT-A/PGS or molecular karyotyping of trophectoderm allows insight into the chromosomal constitution of a blastocyst, but does not confirm the chromosome complement of the future embryo/fetus/neonate, since the trophoblast is foreign to the differentiation of the future embryo that occurs up to 10 days of the embryo transfer or when the blastocyst has nested in the endometrium.

Directed, minimal or expanded carrier genetic testing allows couples to obtain information on whether they carry recessive mutations. Each individual has an estimated five recessive mutations. The problem is when both members of the couple have recessive mutations for the same gene, since one of every four children might inherit the two mutated genes and express the recessive disease; one of every four might not inherit any of the two mutated genes of the parents and might not be affected; and two of the four children might inherit the mutation from one of the parents and neither would be affected. The chances of a non-consanguineous couple having mutations for the same gene, based on the data collected in couples submitted to expanded screening with gametes of their own or from third parties, is estimated at 1/1250. This means that only one in 1249 might benefit from preventive prenatal diagnostic tests. Scientific societies from developed nations have spoken in favor of communicating the possibility of carrying out the above strategies to mitigate the risk of children being born with recessive diseases and chromosome aneuploidies, although without mentioning the cost-effectiveness of the proposition (ACOG, 2017; Gross *et al.*, 2008; Lazarin & Haque, 2016).

## Screening of carriers of recessive mutations or "carrier testing"

The purpose of carrier testing is to find whether asymptomatic couples carry same-gene mutations that place them at 25% risk of having affected children. Every person has an estimated five recessive mutations. Consanguineous couples are more likely to share same-gene mutations.

There are three types of screening:

**Directed:** tests directed to a certain gene on account of family history of recessive disease or to individuals belonging to particular ethnic groups or populations.

**Minimum:** tests evaluating the presence of mutations related to the most frequent diseases in the population or serious diseases that require chronic treatment for life.

**Expanded:** tests designed to find whether couples are carriers of more frequent and/or severe recessive diseases. Commercially available expanded carrier testing kits cover 30, 50, 150, 350 or 650 recessive diseases. Consensus statements published by related scientific societies suggest that mutations need frequencies greater than 1% to express phenotypes that affect quality of life through physical and/or intellectual disability and require chronic medical or surgical care from an early age, and to allow prenatal or pre-implantation stage detection for purposes of live birth optimization.

**Karyotyping:** tests performed through the cultivation of peripheral blood lymphocytes for the study of chromosomes in metaphase. In the population with reproductive disorders, the goal is to detect the presence of balanced structural rearrangements and/or mosaicism with two or more cell lines, generally of sex chromosomes.

## Proportion of children born with congenital malformations

In the general population, three percent of live births are affected by congenital malformations, not all of genetic origin. Half of the defects are of unknown origin. In the realm of classic genetic anomalies, 0.6% of the cases comprise chromosomal abnormalities; 0.4% of the cases are monogenic anomalies (dominant, recessive, and X-linked); and 0.5% relate to polygenic anomalies. Dynamic mutations, mitochondrial and imprinted genetic disorders are very rare. Uncorroborated data indicates that neonates born from ART protocols are estimated to have 30-40% more congenital malformations than the general population of newborns. If the estimation is corroborated, the increase might be due to underlying genetic disorders in the infertile couples or to the procedure per se in any of its stages, either with parent-own or donated gametes. Therefore, in order to minimize risk, couples providing gametes should undergo genetic testing and quality controls should be enforced in all stages of ART procedures.

## Specific risk factors

Individuals carrying dominant mutations have a chance of 50% of transmitting the mutation to their offspring. Since dominant mutations have penetrance and expression variables, the resulting phenotype does not always fully express all features and characteristic symptoms of the mutation.

Subjects with recessive mutations are normal and may transmit the mutation to their offspring without causing inconvenience, unless their partners also carry a mutation for the same gene; in this case, the couple has a 25% chance of having a child affected by a recessive disease.

Women with X-linked recessive mutations are usually normal. Half of their sons may be affected if they inherit the X-linked mutation, whereas half of their daughters are usually normal. Women carrying X-linked dominant mutations have a 50% chance of transmitting the mutation to their children, both male and female. Males with dominant X-linked mutations are at risk of transmitting the disease to their daughters, but not to their sons.

Finally, individuals with polygenic or multifactorial mutations may have affected children if the environment propitiates the expression of the mutation. The inheritance pattern of polygenic mutations is not the same as in monogenic mutations. The risk of recurrence depends on the number of previously affected offspring, with chances at 5% after one affected child and 15% after two. Neural tube defects are examples of multifactorial inheritance. Folic acid supplementation decreases the occurrence of defective closure of the neural tube.

Male carriers of numerical disorders of the autosomes and sex chromosomes are usually sterile for causing meiotic arrest. XYY males can be fertile and have no risk of transmitting two Y chromosomes. Fertile males with the XXY karyotype are mosaics with normal cell lines at the gonadal level or their spermatogonia, before entering meiosis, lost the extra X chromosome, which means they are not at greater risk of generating 24,XX or 24,XY sperm. In contrast, XXX women have a 50% chance of having XXX daughters or XXY sons. Women with free trisomy 21 are also fertile and have a 50% chance of having children with Down syndrome. In contrast, men with Down syndrome are sterile.

Women with karyotype 45,X or with structural abnormalities of the X chromosome usually have rudimentary gonads and primary amenorrhea. Males with structural anomalies of the Y chromosome that imply partial deficiency of the euchromatic domain of the long arm of Yq generally do not produce sperm.

Female and males carrying reciprocal translocations (exchanges of chromosomal segments between non-homologous chromosomes) have a theoretical risk of 80% of producing gametes with chromosomal imbalances due to abnormal segregation of the quadrivalent. The empirical risk almost always matches or is worse than the theoretical risk.

Females and males carrying Robertsonian translocations (centric fusions between acrocentric chromosomes) have a theoretical risk of 75% of producing gametes with chromosomal imbalances by anomalous segregations of the trivalent, but the empirical risk is usually much lower than the theoretical risk and varies according to gender. In male carriers, these translocations are much more benevolent than in women.

Female and male carriers of pericentric inversions (interstitial inversion of a chromosome segment involving the centromere) have a theoretical risk of 66% of producing gametes with chromosomal imbalances due to asymmetrical exchanges during crossing over or nondisjunction during meiosis predisposed by the meiotic chromatin loop. However, the empirical risk is almost always lower than the theoretical risk and depends on the size of the inverted segment and of the involved chromosome.

- Women and men with normal karyotypes may be at risk of producing aneuploid gametes with an extra chromosome or with only one chromosome, either an autosome or sex chromosome. It is well recognized that women with advanced age are more likely to produce aneuploid oocytes, while it is also true that most abnormal embryos with aneuploidy of a whole chromosome are not viable and are lost in preimplantation or in the embryonic stage. Aneuploidies of the autosomes are more lethal than sex chromosome aneuploidies. The rate of aneuploid spermatozoa does not increase with age as it happens with oocytes, but increased dominant mutations occur with paternal aging.
- It is important to note that partial deficiencies or trisomies of the chromosomes are not as lethal as complete deficiencies or trisomies. It is therefore important to rule out the existence of balanced structural rearrangements in the couple, since they would result in greater risk of abnormal offspring.
- Consanguineous couples are more likely to share recessive mutations for the same gene that lead to increased risk of having children with recessive diseases. Certain small populations and ethnic groups with more inbreeding are more likely to carry same-gene mutations.
- Most people carry recessive mutations, but the probability of sharing the same mutations with a partner is very low, unless there is family history in both branches, some degree of kinship or if they belong to certain populations or ethnic groups with a high degree of inbreeding.
- There is not enough data on the probability of a non-consanguineous couple without genetic family history sharing recessive mutations for the same gene. With the implementation of screening for recessive mutations with NGS, the probability has been estimated at 1 in 1250, similar to the risk estimated prior to the advent of NGS at 1/1000 for non-consanguineous couples without family history and not belonging to ethnic groups at greater risk.

With these facts in mind, it should be mandatory to rule out the potential genetic causes of infertility, to evaluate couples physically, and perform complementary studies and pertinent genetic tests.

## What tests should infertile patients undergo prior to attempting to reverse infertility?

Infertile couples with or without a family history should perform the following tests:

1. Couple karyotyping: the prevalence of abnormalities in the general population of newborns is 0.6%; in couples undergoing ART protocols the proportion is 3-5%; in sterile and/or infertile males it ranges from 5% to 15%; and in sterile and/or infertile women it may be as high as 30%. It is very important to know the type of anomaly, since some lead to sterility, while others imply major risk of abnormal offspring. It should be emphasized that numerical autosome and sex chromosome anomalies yield sterility, while balanced structural rearrangements that do not arrest gametogenesis pose a higher risk of producing gametes with partial imbalances of chromosomal segments that are not always lethal, but that lead to birth malformations. Therefore, couples known to carry balanced structural rearrangements are candidates for preventive prenatal diagnosis on account of their increased risk of having malformed newborns; and if they need IVF, they should first undergo PGT-A.
2. AZF microdeletions: Five to ten percent of men with severe oligoasthenoteratozoospermia (OAT) have AZF microdeletions. Knowing the type of microdeletion legitimizes the search for sperm in testicular biopsy to perform ICSI. Deficiencies in areas AZFa, AZFb and AZFc as well as AZFa and AZFa+AZFb alterations are contraindications to performing biopsy, since these microdeletions are pathognomonic of absence of germinal epithelium. Males with AZFc microdeletion are able to produce sperm, and their sons will have the same deleted region and inherit infertility from their fathers.
3. Fragile X premutation: Twenty to thirty percent of women with premature ovarian failure (POF) have expansion of the CGC triplet repeat within the FMR1 gene (50 to 200 repeats) and are at higher risk of having sons with mental retardation, since this is a dynamic mutation that expands every generation. Expansions exceeding 200 repeats are categorized as full mutations. Males with the full mutation suffer with mental development delays and are characterized as expressing the Martin Bell syndrome, while 10% of the females with the mutation may have delayed mental development. Couples with expansion of the CGC triplet in need of IVF are candidates for prenatal diagnosis or PGT-M.
4. Cystic fibrosis mutations: Twenty-two percent of the males with oligozoospermia carry mutations of the CFTR gene; 30% present with cryptozoospermia and 80% with azoospermia due to bilateral agenesis of the vas deferens; 20% carry one mutation, 20% two mutations, 30% one mutation + one 5T variant, and 10% have 5T variants. One might say that it is almost mandatory to screen males with agenesis of the vas deferens and, depending on test results, extend the studies to their wives. If the two are carriers, their chance of having severely affected children with cystic fibrosis resides between 25% and 50%. Since there are more than a thousand mutations of the CFTR gene, ideally complete sequencing should be performed by NGS in the male partner and the tests extended to the female if he tests positive.

## What other tests may be offered to couples apparently without genetic disorders associated with infertility?

Couples without genetic disorders associated with infertility should be informed that at present there are different genetic tests that allow the search for recessive mutations that might lead to increased risk of having affected children if both carry mutations for the same gene. Most individuals carry between 1 and 10 recessive mutations that might be transmitted to half of their offspring without much problem. The issue appears when both members of the couple share mutations for the same gene. Although it was mentioned above that this possibility is minimal, those interested in radically minimizing the chances of having children affected by recessive diseases may choose to undergo a wide array of carrier genetic tests. One of the members of the couple may be screened first, and if the individual is found to be mutation-free, his or her partner does not have to be tested. However, if the tested partner has a mutation in a particular gene, the other partner should be tested for mutations on that same gene. When using her own oocytes and donor sperm, the recipient may be tested, and if she is negative for the tested mutations, the sperm donor does not have to be tested. On the other hand, when donor oocytes are used, it is prudent to screen the male partner and test the egg donor for X-linked mutations. This way less is spent with testing and the disclosure of genetic data from donors would be limited; although donors are paid, the act of donating is inherently altruistic and worthy of respect and gratitude.

## The benefits of helping patients make informed decisions

In order to make the best use of their procreative freedom, couples with and without family history of genetic disease should be provided genetic counseling with a specialist. In addition, couples must understand that testing does not guarantee a normal gestation, so once they become pregnant they should be informed of the convenience of pregnancy follow-up and non-invasive or invasive prenatal genetic tests in cases suspected for genetic anomalies. Since not all preconception studies are covered by private or public health insurance, patients unable to afford them should be aware of the risks of having children with recessive conditions or aneuploidies. The risk-benefit ratio of new preconception tests for couples undergoing ART procedures is still unclear; therefore, it is advisable to discuss with a geneticist the chances of having children with genetic diseases. Only after understanding the magnitude of the risk, each person, according to his or her beliefs and possibilities, should decide whether or not to undergo preconception testing.

## What other alternatives do couples have?

1. Preimplantation genetic testing in its two versions: PGT-M and PGT-A
  Couples requiring IVF/ICSI to achieve pregnancy may have blastocysts tested prior to transfer. PGS, now called PGT-A, is the study of the molecular karyotype of the trophoblast, which allows inferences on the chromosomal constitution of the blastocyst, but may not correspond with the karyotype of the future embryo-fetus-newborn due to the fact that embryo differentiation starts when the blastocyst has attached to the endometrium. PGD, now called PGT-M, is a prenatal diagnostic test performed when there is risk of a particular monogenic condition occurring. The purpose of PGT-A/PGS is to allow the transfer of euploid embryos, which are more likely to originate ongoing pregnancies and thus a greater number of live births. Nevertheless, the evidence around it is still lacking. In couples with good prognosis, systematic reviews have shown a significant increase in the rates of ongoing pregnancies per transfer, but not per cycle performed. Therefore, they should be informed that the PGT-A might not increase the rate of live births per initiated cycle, but rather decrease it. In addition, there is a greater possibility that the couple may not undergo the transfer procedure because all blastocysts may be abnormal. However, a PGT-A test result showing an aneuploidy does not necessarily mean that the transfer cannot occur, since an estimated 4% of aneuploid blastocysts originate normal newborns (Gleisher *et al*., 2015; Greco *et al*., 2015; Orvieto, 2016). According to the recommendations of the Preimplantation Genetic Diagnosis International Society (PGDIS) (PGDIS Newsletter, 2016), monosomies, with the exception of the ones affecting the sex chromosomes, do not preclude transfer; only trisomies that do not produce disease in newborns and do not involve chromosomes with imprinted genes might be considered, since uniparental disomies stemmed from trisomic rescue might cause imprinting diseases, such as trisomies 14 and 15; although confined to the placenta, trisomies 2, 7 and 16 should be avoided, since they are associated with marked growth retardation. Although it is true that the rate of aneuploidy fertilization is important, even in young couples carrying normal karyotypes, most of them are numerical anomalies of whole chromosomes, 99% of which lethal during preimplantation and early embryo- fetal development. After achieving pregnancy, with or without PGT-M/PGT-A, it is always advisable to confirm the results of PGT with different non-invasive or invasive prenatal tests, of which amniocentesis provides for higher diagnostic certainty.
2. Non-invasive prenatal tests using maternal blood to screen for aneuploid pregnancies.
3. Study of ultrasound markers in week 11 together with biochemical screening on the first and second trimesters of pregnancy.
4. Prenatal diagnosis by chorionic villus puncture.
5. Prenatal diagnosis in amniocytes from amniotic fluid.
6. Genetic study of umbilical cord blood in the second trimester of pregnancy or at the time of delivery.

Not all the congenital disorders are of genetic origin. They may have uncertain origins or be linked to factors such as maternal exposure during pregnancy to chickenpox, rubella, toxicants, certain drugs or lack of folic acid or malnutrition. These anomalies may be minimized if recommended preventive measures are enforced, such as immunization against rubella and chickenpox, sufficient intake or supplementation with folic acid and iodine, in addition to prenatal care for non-exposure to teratogenic agents.

It should be noted that each person is the owner of his or her genetic code. Genetic counseling is optional, not compulsory. The indication of performing genetic tests should not be prescriptive or coercive.

## Ethical and legal considerations

Before the start of ART treatment, individuals and couples should give written consent confirming that they were informed about preconception genetic counseling, screening for recessive mutations, preimplantation genetic testing for aneuploidies or monogenic mutations in couples at increased genetic risk, and that diagnostic tests are not perfectly accurate and that other prenatal diagnostic tests are available and may be performed during pregnancy.

After having been informed of the possible genetic risks and having understood the limitations of every evaluation test, they must choose from the proposed tests and consent to being tested, knowing that the main limitation is that no genetic test can guarantee that the newborn will not have congenital malformations, genetic or not in origin.
